# Carbapenem Versus Non-carbapenem Therapy in Hematology Patients: Extended-Spectrum Beta-Lactamase Positive Enterobacteriaceae Colonization Impact

**DOI:** 10.7759/cureus.63570

**Published:** 2024-07-01

**Authors:** Hande Berk, Nefise Oztoprak, Filiz Kizilates, Erdal Kurtoğlu, Aysegul Seremet Keskin

**Affiliations:** 1 Infectious Diseases and Clinical Microbiology Clinic, Antalya Education and Research Hospital, Antalya, TUR; 2 Infectious Diseases and Clinical Microbiology Clinic, Anatolia Hospital Lara, Antalya, TUR; 3 Hematology Clinic, Antalya Education and Research Hospital, Antalya, TUR

**Keywords:** rectal colonization, hematology, mortality, emprical antibiotherapy, esbl

## Abstract

Background: Extended-spectrum beta-lactamase-producing *Enterobacteriacea *(ESBL-E) infections are a major source of mortality and morbidity in patients with hematologic cancers. One of the most significant risk factors for bacterial illness is prior colonization with resistant germs. Empiric usage of carbapenems is recommended for the treatment of infections in patients with a positive colonization history.

Objectives: We aimed to determine the outcome of empirical carbapenem (de-escalation) versus non-carbapenem (escalation) therapy in adult hematology patients who have rectal extended-spectrum beta-lactamase positive ESBL-E colonization.

Methods: Two hundred three rectal swab cultures were collected from 130 patients, admission or during hospitalization between June 2014 and May 2015. Patients were followed till January 2016 for future infections due to ESBL-E. Empirical antibiotic treatment was started according to the patient’s medical condition without consideration of previous colonization status. Stable patients received empirical escalation therapy. All-cause and early mortality of infections are analyzed.

Results: Seventy-three (36%) swabs were positive for ESBL-E. Patients with rectal ESBL-E colonization were defined as cases; patients without colonization were defined as controls. Prospective infection due to ESBL-E in the case and control group was 6.8% and 2.3%, respectively. No statistically significant relation was found between colonization and prospective infection due to ESBL-E (p=0.110). There was no all-cause or early mortality in prospective infections with ESBL-E. Among case patients, one patient each died from all-cause mortality in the escalation (n=55) and de-escalation (n=3) group. The all-cause mortality in the antibiotic switch group (n=30) was eight, including five patients in the early mortality group although the bacteriologic agents were susceptible to the given antibiotics.

Conclusion: In our institution, rectal colonization with ESBL-E was high, but contracting an infection due to ESBL-E was surprisingly low. Colonization with ESBL-E may not necessarily end with an infection in some situations. In stable patients, disregarding colonization features before empirical therapy did not seem to be inappropriate.

## Introduction

Infections due to extended-spectrum beta-lactamase-producing *Enterobacteriacea* (ESBL-E) are an important cause of mortality and morbidity in patients with hematologic cancer. Studies show that inappropriate coverage of empiric antibiotic therapy in these infections significantly and independently impairs outcomes, increasing mortality, and prolonging hospitalization [1,2].

One of the major risk factors for infection with resistant bacteria is the patient’s prior colonization or infection with resistant microorganisms. In case of colonization or previous infection with resistant bacteria, de-escalation therapy (DET) with the usage of carbapenems in the empirical therapy is recommended for febrile neutropenic (FEN) patients anticipated to have neutropenia for more than seven days [1-4]. Systematic screening of patients’ gastrointestinal colonization on admission and once or twice weekly is also suggested in those centers with dense frequencies of resistant pathogens [5-7].

In this study, we aimed to determine the risk factors for rectal colonization with ESBL-E in our adult hematology clinic and the impact of carbapenem versus non-carbapenem-based empirical therapy on the outcomes of FEN and febrile episodes (FE) in patients who have rectal ESBL-E colonization or not.

## Materials and methods

Setting, patient selection, and collection of rectal samples

The study was conducted in a 24-bed capacity adult hematology clinic of a 900-bed tertiary referral hospital. Patients who are ≥18 years old with or without any hematological malignancy admitted to our hematology clinic between June 2014 and May 2015 were enrolled in the study. Rectal swabs were collected from the patients either on admission or during the hospitalization period. In each hospitalization period, only one rectal swab was collected from the same patient. Repeat rectal swabs were taken from the patients who allowed the collection procedure. To determine the impact of non-carbapenem-based escalation therapy (ET) on the outcomes of the infections of patients with positive rectal ESBL-E colonization; these patients were followed throughout their hospitalization periods for prospective infections till January 2016 or the patient’s death. During the same study period, future infections due to ESBL-E in non-colonized patients were also noted. Patient demographics, possible risk factors for colonization, empirical antibiotic therapy, infection type, etiological agents with culture results, and outcome of the infections were recorded. The study was approved by our local ethical committee (19/06/2014-44/17) and informed consent was taken from the study patients.

Antibiotics

In the study, if the patient’s status was stable, a non-carbapenem-based ET during FEN episodes according to the 2011 Infectious Diseases Society of America (IDSA) guideline was carried out [8]. As an empirical therapy, FEN patients received intravenous (IV) therapy with anti-pseudomonal beta-lactams including piperacillin-tazobactam 4.5 g q6h, imipenem/cilastatin 500g q6h or meropenem 1g q8h as a monotherapy or with a combination of aminoglycoside therapy as soon as and after relevant cultures were drawn from the patient. No prophylactic antibiotic therapy was given to our patients during the chemotherapy period. In non-neutropenic patients, empirical antibiotic therapy was started with ET unless the patient was in a septic condition.

Definitions

**Figure 1:**
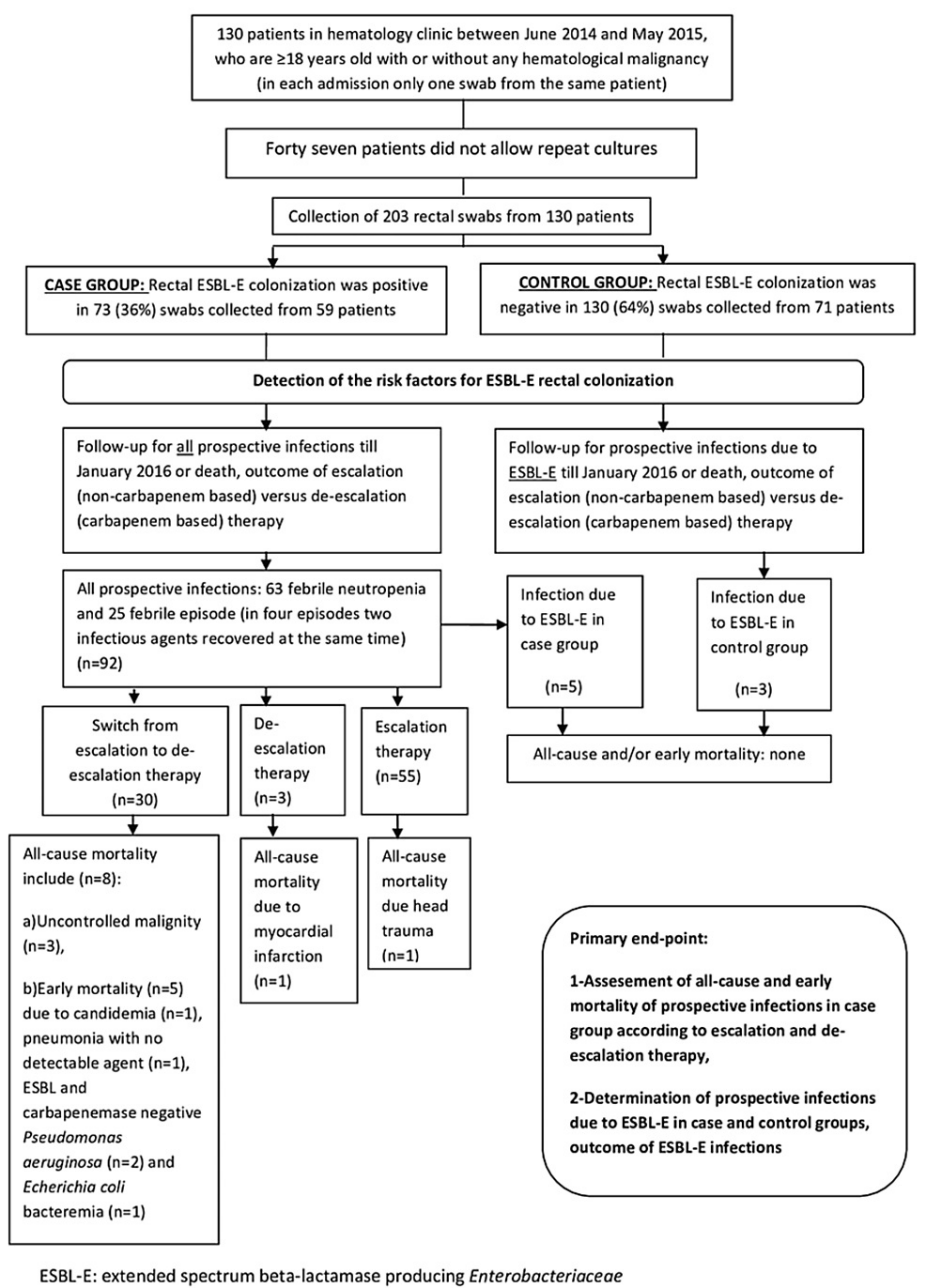
Flow chart of the study ESBL-E: extended-spectrum beta-lactamase producing *Enterobacteriaceae*

Patients with rectal ESBL-E colonization were recorded in the case group and patients without colonization were recorded in the control group. FE is defined as a patient with a fever of 38°C or higher that persisted for two to seven days with or without a localizing source of infection and an FEN episode is defined according to 2011 IDSA guidelines [8]. In a FE or FEN episode, the start of initial empirical antibiotic therapy with carbapenems was defined as “de-escalation therapy” and the start of initial antibiotic therapy other than carbapenems was defined as “escalation therapy.” A switch from ET to DET was implemented in case of a worsening condition. Rectal swabs collected within 48 hours of admission were defined as “on-admission” but collection more than 48 hours were defined as “nosocomial.” All-cause mortality is defined as mortality due to any cause within 28 days whereas early mortality is defined as mortality observed during the following seven days after the start of an empirical antibiotic therapy. The primary endpoints were assessment and comparison of the rate of all-cause and early mortality in the case group treated with either ET or DET and analysis of the outcome of ESBL-E infections in case and control groups throughout the follow-up period. The flowchart of the study is shown in Figure 1.

Microbiology

Rectal swabs taken from the patients were plated directly onto HiChrome ESBL agar base medium (BioMérieux, Lyon, France) which is a chromogenic screening medium for the selective isolation of ESBL-producing organisms. Peptone mix and yeast extract serve as the carbon and nitrogen sources. Differentiation of the ESBL-producing bacteria is made by a chromogenic mixture on the basis of color. After 48 hours of incubation at 35°C and under aerobic conditions, the colors of the colonies were assessed for ESBL production according to the color chart provided by the manufacturer. At least three oxidase-negative colonies, positive for ESBL production were selected and the Vitek-2 microbial identification system (BioMerieux, Marcy l’Etoile, France) was used for the identification of the bacteria. Confirmation of ESBL production was performed by Kirby-Bauer disc diffusion method and 30 µg ceftazidime versus 30/10 µg ceftazidime-clavulanate and 30 µg cefotaxime versus 30/10 µg cefotaxime-clavulanate discs were used for detection of ESBL enzyme according to the Clinical and Laboratory Standards Institute criteria. *Escherichia coli* ATCC 25922 and *Klebsiella pneumoniae *ATCC 700603 were used as controls. Identification of the species level and antibiotic susceptibilities of the bacterial agents recovered from the patients during FE or FEN episodes in the follow-up period were also studied with Vitek-2 according to CLSI criteria.

Contact precautions

All patient rooms are composed of two beds and a shared bathroom. Contact precautions were implemented for the patients confirmed with ESBL infection but contact precautions could not be implemented for the patients with rectal ESBL-E colonization due to inadequate bed capacity.

Statistical analysis

Statistical analysis was performed using SPSS software version 18.0 (IBM Corp., Armonk, NY, USA). The relationship between categorical variables was measured by the Pearson chi-square test. The odds ratio (OR) with 95% confidence intervals (CI) was calculated. Student T and Mann-Whitney U tests were used to compare the differences between the two groups. Multiple binary logistic regression analysis was performed for the effects of variables. Receiver operating characteristic (ROC) analysis was performed for the age variable. P-value < 0.05 was accepted as significant.

## Results

Two hundred three rectal swab cultures were taken from 130 patients. The mean age was 53.82±17.11 years and 59.1% was male. Among 203 rectal swabs, 73 (36%) swabs taken from 59 patients were positive for ESBL-E. Forty-seven patients who had rectal ESBL-E colonization did not permit repeat rectal cultures due to ethical considerations like social isolation but in the remaining 12 patients, rectal swabs were collected from two patients three times and from 10 patients twice. On-admission colonization rate was 28% (14/50) and nosocomial colonization rate was 38.6% (59/153). There was no statistically significant relationship found between on-admission and nosocomial rates of rectal ESBL-E colonization (p=0.245). Only patients aged≥55 were found as a risk factor for ESBL-E colonization (p=0.001, OR: 2.80, 95%CI: 1.51-5.16). The main baseline and demographic characteristics of case and control groups in the study population are shown in Table 1.

**Table 1:** Baseline characteristics of the study population (a) Statistically significant. (b) Receiver operation curve analysis is performed for categorical analysis of the age variable according to clinician’s decision. DM: diabetes mellitus, COPD: chronic obstructive pulmonary disease, BPH: benign prostatic hypertrophy, UTI: urinary tract infection, CVC: central venous catheter, ESBL-E: extended spectrum beta-lactamase producing *Enterobacteriaceae*

Characteristics	Case (%) n: 73 (36%)	Control (%) n: 130 (64%)	P-value OR (95% CI)
Male gender (n, %)	45 (61.6)	75 (57.7)	0.583
Age (Mean, SD)	56.89, ±15.31	52.10, ±17.86	0.046^a^
Age≥55^b^	52 (71.2)	61 (46.9)	0.001^a^ 2.80 (1.51-5.16)
Underlying disease (n, %)			
Hematologic malignity	65 (89.0)	114 (87.6)	0.901
Acute leukemia	19 (26)	44 (33.8)	
Lymphoma	20 (27.4)	43 (33.1)	
Multiple myeloma	22 (30.1)	17 (13.1)	
Myelodysplastic syndrome	3 (4.1)	5 (3.8)	
Myeloproliferative disease	1 (1.4)	5 (3.8)	
Autoimmune hemolytic anemia	7 (9.6)	10 (7.7)	0.192
Thalassemia disease	1 (1.4)	6 (4.6)	0.123
DM	4 (5.5)	13 (10)	0.265
Renal failure	15 (20.5)	18 (13.8)	0.214
COPD	6 (8.2)	15 (11.5)	0.456
Nephrolithiasis	14 (19.2)	14 (19.2)	0.993
BPH in male patients	4 (8.8)	11 (14.6)	0.258
UTI in last year	12 (16.4)	20(15.4)	0.843
Hospital admission ≥48 hours in six months	58 (79.5)	94 (72.3)	0.260
Surgery in six months	17 (23.3)	27 (20.8)	0.676
Endoscopy, colonoscopy in six months	9 (12.3)	17 (13.1)	0.878
CVC in 6 months	25 (34.2)	34 (26.2)	0.223
Urinary catheter in six months	12 (16.4)	17 (13.1)	0.511
Immunosuppressive therapy	48 (65.8)	70 (53.8)	0.099
Usage of antibiotic in six months	56(76.7)	87 (66.9)	0.142
Usage of antibiotic in 30 days (n, %)	43 (58.9)	64 (49.2)	0.185
Day of swab culture since admission in days Median, min-max/mean ± SD	5, 0-57 9.92±12.24	5, 0-65 7.62±9.37	0.268
Prospective ESBL-E infection	5 (6.8)	3 (2.3)	0.110

Among 73 ESBL-E positive rectal swabs, *E. coli *was detected in 56 (27.6%) samples, *K. pneumoniae* in eight (3.9%) samples, and *Klebsiella oxytoca* in two (0.9%) samples. In seven samples (3.4%),* E. coli *and *K. pneumonia* were recovered from the same rectal swab at one time.

Fifty-nine patients who have rectal ESBL-E colonization (n=73) were prospectively followed till January 2016 or death in terms of FE and FEN episodes. The median time of follow was 300 days (minimum: 15, maximum: 550 days, mean: 280 ± 171.7 days). In 27 patients (45.7%), no FE and FEN were detected during the follow-up period (mean: 271.4 ±179.6 days) while in the remaining 32 patients, 63 FEN episodes, and 25 FE were detected. An etiologic agent or infectious foci could not be found in 30 episodes, but bloodstream infection (BSI) was the most predominant infection (Table 2).

**Table 2:** Type of prospective documented infections in case patients (a) Carbapenem-resistant isolates were *E. coli* isolates which were ESBL-negative (b) In four episodes, concomitant secondary bacteremia due to the primary infectious focus resulted in the recovery of two microorganisms at one time ESBL: extended-spectrum beta-lactamase, n.a: not-applicable, CVC: central venous catheter

	Bloodstream infection (n=26)	Pneumonia n=16	Urinary tract infection n=5	CVC-related bloodstream infection n=5	Intra-abdominal infection n=4	Skin and soft tissue infection n-=6	Total documented infection ^b^ n=62
Microbiologically documented infection	26 (100%)	9 (56.2%)	5 (100%)	5 (100%)	-	4 (66.6%)	49 (79%)
ESBL positive infection	3 (11.5%)	-	2 (40%)	-	-	-	5 (8%)
Carbapenem-resistant infection ^a^	1 (3.8%)	-	-	-	-	1 (16.6%)	2 (3.2%)
Methicillin-resistant infection	-	-	-	5 (100%)	-	-	5 (8%)
Clinically documented infection	n.a	7 (43.7%)	-	n.a	4 (100%)	2 (33.3%)	13 (21%)

There was a tendency toward gram-negative bacteria recovered from FEN or FE in the case group*. E. coli* was the most frequent pathogen (n=25, 50%) followed by *Pseudomonas aeruginosa* (n=8, 16.1%) (Table 3). Among gram-negative microorganisms, ESBL was detected in five isolates which were all *E. coli* isolates recovered from BSI (n=3) and UTI (n=2) (Table 3).

**Table 3:** Infectious agents recovered from the case patients (a) Carbapenem-resistance was detected in only two *E. coli* isolates excluding *S. maltophilia* which is genetically resistant (b) Among gram-negative microorganisms ESBL was detected in only five *E. coli* isolates (c) Includes central venous catheter related bloodstream infections

Microorganism	Total, n (%)	Bloodstream infection^c ^, n (%)
Gram negative microorganisms ^a^		
*Escherichia coli *^b^	25 (50)	15 (48.3)
Pseudomonas aeruginosa	8 (15.9)	5 (16.1)
Klebsiella pneumoniae	2 (4.5)	2 (6.4)
Stenotrophomonas maltophilia	2 (4.5)	-
Acinetobacter baumanii	2 (4.5)	-
Enterobacter aerogenes	1 (2.3)	1 (3.2)
Gram positive microorganisms		
Methicillin resistant coagulase-negative staphylococci ^c^	5 (11.3)	5 (16.1)
Enterococcus faecalis	1 (2.3)	-
Streptococcus pneumoniae	1 (2.3)	1 (3.2)
Candida albicans	2 (2.3)	2 (6.4)
Total	n=49 (100)	n=31 (100)

Prospective infection due to ESBL-E in the case and control group was 6.8% (n=5) and 2.3% (n=3), respectively. There was no statistically significant relation between colonization with ESBL-E and prospective infection due to ESBL-E (p= 0.110). The eight patients who had a prospective infection caused by ESBL and the outcomes are shown in Table 4.

**Table 4:** Outcome of prospective infections due to extended spectrum beta-lactamase producing Enterobacteriaceae in case and control groups M: male, F: female, ESBL-E: extended-spectrum beta-lactamase-producing *Enterobacteriaceae*, NHL: non-Hodgkin Lymphoma, ALL: acute lymphoblastic leukemia, AML: acute myeloid leukemia, CLL: chronic  lymphocytic leukemia, MM: multiple myeloma, APSCT: autologous peripheral stem cell transplantation, FEN: febrile neutropenia, TZP: piperacillin-tazobactam (4.5 g q6h), MIC: minimum inhibitory concentration, EC: *Escherichia coli,* KP: *Klebsiella pneumoniae*, MEM: meropenem (meropenem 1g q8h)

ESBL-E infection (n, %)	Patient	Year, Gender (M/F)	ESBL-E colonization	Hematologic disease	Episode and infection type	Organism, length of time between recovery of the microorganism and rectal swab collection, TZP MIC value	Empirical antibiotic therapy, reason	De-escalation to carbapenem due to inadequate therapy	Mortality (all-cause/early)
Control group n:3, (2.3%)	1	38 y, ( M)	None	NHL (refractory terminal disease with bulky intra-abdominal tumor, supportive care)	FEN, Pneumonia	EC, 18 days, ≤4/4	MEM, recent 10 days of TZP usage due to infection secondary to intra-abdominal bulky tumor,	-	None
	2	44y,( M)	None	ALL (consolidation chemotherapy)	FEN, UTI	EC, 13 days, ≤4/4	TZP	None	None
	3	80y, (F)	None	AML (supportive care)	FEN, UTI	EC, 23 days, ≤4/4	TZP	None	None
Case group n:5 (6.8%)	4	69 y, (F)	Yes	AML (induction chemotherapy)	FEN, bloodstream infection secondary to UTI	KP, 5 days, ≤4/4	TZP	Although symptoms and signs resolved with TZP, therapy was shifted to meropenem due to concomitant bacteremia on day 4 of FEN	None
	5	69y, (M)	Yes	CLL(induction chemotherapy)	FEN, UTI	EC, 23 days, ≤4/4	TZP	Although fever resolved with TZP, therapy was shifted to meropenem due to ongoing symptoms on day 4of FEN	None
	6	59, (F)	Yes	ALL (induction chemotherapy)	FEN,UTI	EC, 23 days, >64/4	TZP	Fever and symptoms did not resolve with TZP, therapy was shifted to meropenem on day 2 of FEN	None
	7	62, (M)	Yes	MM (APSCT)	FEN, Bacteremia	EC, 55 days, ≤4/4	MEM, the patient was already receiving 10 days of TZP when FEN occurred	-	None
	8	52, (M)	Yes	MM (APSCT)	FEN, Bacteremia	EC, 125 day, ≤4/4	TZP	Fever and symptoms did not resolve with TZP, therapy was shifted to meropenem on day 2 of FEN	None

At the end of the study period, 10 patients (n=73) in the case group died due to all-cause mortality (13.6%) including five patients who died because of early mortality (6.8%). For those five patients (one FE patient and four FEN patients) switch from escalation to DET was needed but the patients could not survive although the etiologic bacterial agents (one *E. coli* and two *Pseudomonas aeruginosa* isolates) were ESBL negative and susceptible to piperacillin-tazobactam (TZP) and carbapenems (Figure 1).

## Discussion

One of the main risk factors for infection by ESBL-E is the patient’s gut colonization which is an important reservoir [1,8-10]. Before 2008, the global prevalence of ESBL-E in the fecal samples of the community was below 10%, but in 2008 a report from Thailand increased the prevalence to 58.2% [11]. A new study from our country including patients who were to undergo transrectal ultrasonography-guided prostate biopsy reported on-admission ESBL-E colonization as 19% [12]. Recently, a notable rate of rectal ESBL-E colonization of up to 21.5% was found in healthy children admitted to our hospital for circumcision procedure [13]. In the present study, the on-admission rectal colonization rate was 28% whereas the nosocomial colonization rate was 38.6% with no statistically significant difference (p=0.245) which might be because our patients had already multiple co-morbidities or healthcare-associated implementations on admission (Table 1). Consistent with our findings, studies with hematologic patients report similar rates of on-admission and nosocomial ESBL-E colonization up to 14.3% and 17.5%, showing no significant difference between [14,15].

In the study, there was no significant association between the duration of hospitalization (the time of rectal swab collection since admission in terms of days) and ESBL-E colonization (p= 0.268) (Table 1). This was an interesting finding because moderate evidence suggests contact isolation precautions for both infected and colonized patients [16]. However, during the study period isolation of patient rooms could not be performed due to the inadequacy of bed capacity. Additional data show that the median time of clearance of rectal ESBL-E colonization after discharge from the hospital may be as long as 6.6 months [17,18]. Assuming the similar length of time for clearance for ESBL-E colonization in the patients, together with an admission colonization rate of 28%, nosocomial ESBL-E colonization rate of 38.6%, and no contact precaution implementation to the colonized patients; patient to patient transmission of ESBL-E from colonized patients to the non-colonized patients did not seem to make a statistical difference in our ESBL-E colonization rates while the following implementations were being conducted during the study period: a) regular education program about infection control measures for patients, their caregivers and healthcare staff, b) hand hygiene compliance of the healthcare staff which was around 76.4% (unpublished data), c) patient daily oral care and patient bathing at least three times a week, and d) regular environmental cleaning procedures implemented by the same cleaning staff. The present finding was against most international guidelines which suggest contact precautions to the colonized patients but, until new data are gathered, we do recommend contact precautions to the colonized patients [16,19].

Although there are numerous reports regarding previous antibiotic usage as a strong risk factor for ESBL infection or colonization there was no statistically significant relation between previous antibiotic usage and ESBL-E colonization despite the high frequency of antibiotic usage (p=0.142) [14,20-22]. Since further classification of the antibiotics was not performed, the impact of the usage of different antibiotic classes could not be demonstrated (Table 1).

In the literature risk factors associated with ESBL-E colonization other than previous antibiotic usage include age≥75 years, male gender, prior hospitalization, underlying disease, mechanical ventilation, and recent surgery but in the present study, only age ≥55 years was found as a risk factor for ESBL-E colonization (OR: 2.80, 95% CI:1.51-5.16) [20,21].

We detected a high rate of rectal ESBL-E colonization which was 36% but the rate of prospective infection due to ESBL-E was 6.8% (n=5) in this group. Also, subsequent BSI due to ESBL-E was only 2.3% (n=3) (Table 2). In hematology and oncology units, Liss et al. reported intestinal colonization with ESBL-E and subsequent BSI as 17.5% and 6.6%, whereas Reddy et al. reported the rate as 4% and 8.5%, respectively [23,24]. However, our rate of subsequent BSI due to ESBL-E in colonized patients was lower than other study results despite high rates of colonization. We could not perform molecular typing or pulse gel field electrophoresis to prove intestinal transmission of these virulent isolates to the sterile tissues which was a limitation of our study. To our knowledge, there are no studies regarding the quantitative percentage of these ESBL-positive bacteria in the intestinal flora of the patients and the threshold intestinal bacterial load that may lead to an infection. Besides, although there are numerous studies regarding the virulence factors of ESBL-E strains; there is an urgent need for a better understanding of the pathogen load and interaction of these virulence factors with different hosts having diverse epidemiological and underlying features such as neutropenia [25,26].

In the literature regular screening of the patients on admission and once or twice weekly is suggested in centers with a high prevalence of antibiotic resistance [1,16]. Also, previous colonization with resistant bacteria is accepted as one of the factors for choosing DET during an FEN episode due to the high mortality of inappropriate antibiotic therapy [1]. However, in the present study, unexpectedly low rates of subsequent infection despite the high rate of rectal ESBL-E colonization showed that finding positive intestinal colonization in this patient group does not always necessitate empirical DET in some settings.

In a cohort study comparing the 14-day mortality between TZP and carbapenem as the empiric therapy for 331 ESBL bacteremia, TZP usage 4.5 g q8 (IV) was associated with a higher rate of death than carbapenem therapy (the adjusted risk of death: 1.92 (95% CI: 1.07-3.45) but the authors reported that in a small number of group of patients in which TZP was used as 4.5 g q6 (IV), no statistical significant difference was found in mortality rates versus carbapenem therapy [27]. In the present study, there was no all-cause and early mortality due to an infection caused by ESBL-E in case and control groups receiving either ET or DET. All patients were FEN patients receiving a TZP dosage of 4.5 g q6 and excluding one patient all isolates were susceptible to TZP (Table 4).

Oztoprak et al. stated that empirical TZP or carbapenem treatment with or without aminoglycoside combination therapy was equally effective in FEN patients and no difference was found regarding rude mortality [28]. In another meta-analysis comparing anti-pseudomonal beta-lactam antibiotics in FEN patients, TZP was associated with lower mortality, therefore, the authors suggest empirical usage of TZP in centers with low resistance profiles [28]. Finally, in a recent study comparing the outcomes of empirical therapy in 427 FEN patients, it was concluded that unless there is evidence of sepsis and septic shock, empirical carbapenem therapy may be unnecessary regardless of previous microbiology results [1].

Limitations of the study

In our study, no significant difference in days was found between the times of rectal swab culture collection post-admission for the case and control groups. However, a limitation of the study is that only a single rectal swab culture was collected per admission. Additionally, no concurrent rectal swabs were taken during febrile neutropenia episodes to reflect the current colonization status of the patients.

Among ESBL-E positive cases, the number of patients receiving empiric carbapenem therapy (de-escalation group) was low. Consequently, we could not comment on whether there was a difference in mortality between this group and those who received empiric non-carbapenem-based therapy (escalation group). Furthermore, the mortality rates between the case and control groups with and without ESBL-E-positive colonization could not be compared due to the insufficient number of ESBL-E-positive infections in each group. Future studies with larger patient cohorts are needed to achieve statistically significant results in this regard.

## Conclusions

In our institution, rectal colonization with ESBL-E was high, but contracting an infection due to ESBL-E was low, highlighting the urgent need for better predictive markers for the etiologic agents of prospective infections other than ESBL-E colonization status, making the subject of pathophysiological mechanisms in human gut flora leading to invasive infection in different hosts, future areas for investigation. Disregarding colonization features before the start of an empirical therapy may not be inappropriate in patients who are stable in this special population preventing unnecessary carbapenem prescription in the era of growing antibiotic resistance.
